# Supplementary material for: Using high-resolution melting to identify Calliphoridae (blowflies) species from Brazil
Source: PeerJ. 2020 Nov 30;8:e9680. doi: 10.7717/peerj.9680 (PMC7713596; doi:10.7717/peerj.9680)
Supplement: Supplemental Information 1 [file peerj-08-9680-s001.docx]

**Supplementary Table 1.** Calliphoridae species samples used in this study.

| **Code** | **Specie** | **Source** | **Material** |
| --- | --- | --- | --- |
| Lc1 | *Lucilia cuprina* | Campinas - SP | Fresh |
| Lc2 | *Lucilia cuprina* | Campinas - SP | Fresh |
| Lc3 | *Lucilia cuprina* | Campinas - SP | Fresh |
| Lc4 | *Lucilia cuprina* | Campinas - SP | Fresh |
| Lc5 | *Lucilia cuprina* | Campinas - SP | Fresh |
| Le1 | *Lucilia eximia* | Rio de Janeiro - RJ | Dry |
| Le2 | *Lucilia eximia* | Rio de Janeiro - RJ | Dry |
| Le3 | *Lucilia eximia* | Rio de Janeiro - RJ | Dry |
| Le4 | *Lucilia eximia* | Rio de Janeiro - RJ | Dry |
| Le5 | *Lucilia eximia* | Rio de Janeiro - RJ | Dry |
| 199 | *Lucilia eximia* | Alegre - ES | Alcohol 70 ºG.L. |
| Cme1 | *Chrysomya megacephala* | Alegre - ES | Alcohol 70 ºG.L. |
| Cme2 | *Chrysomya megacephala* | Alegre - ES | Alcohol 70 ºG.L. |
| Cme3 | *Chrysomya megacephala* | Alegre - ES | Alcohol 70 ºG.L. |
| Cme4 | *Chrysomya megacephala* | Alegre - ES | Alcohol 70 ºG.L. |
| Cme5 | *Chrysomya megacephala* | Alegre - ES | Alcohol 70 ºG.L. |
| Cme6 | *Chrysomya megacephala* | Alegre - ES | Alcohol 70 ºG.L. |
| Cme7 | *Chrysomya megacephala* | Alegre - ES | Alcohol 70 ºG.L. |
| Cme8 | *Chrysomya megacephala* | Alegre - ES | Alcohol 70 ºG.L. |
| Ca1 | *Chrysomya albiceps* | Alegre - ES | Alcohol 70 ºG.L. |
| Ca2 | *Chrysomya albiceps* | Alegre - ES | Alcohol 70 ºG.L. |
| Ca3 | *Chrysomya albiceps* | Alegre - ES | Alcohol 70 ºG.L. |
| Ca4 | *Chrysomya albiceps* | Alegre - ES | Alcohol 70 ºG.L. |
| Ca5 | *Chrysomya albiceps* | Rio de Janeiro - RJ | Dry |
| Ca6 | *Chrysomya albiceps* | Rio de Janeiro - RJ | Dry |
| Ca7 | *Chrysomya albiceps* | Rio de Janeiro - RJ | Dry |
| Ca8 | *Chrysomya albiceps* | Rio de Janeiro - RJ | Dry |
| Cp1 | *Chrysomya putoria* | Alegre - ES | Alcohol 70 ºG.L. |
| Cp2 | *Chrysomya putoria* | Alegre - ES | Alcohol 70 ºG.L. |
| Cp3 | *Chrysomya putoria* | Alegre - ES | Alcohol 70 ºG.L. |
| Cp4 | *Chrysomya putoria* | Alegre - ES | Alcohol 70 ºG.L. |
| Cp5 | *Chrysomya putoria* | Rio de Janeiro - RJ | Dry |
| Cp6 | *Chrysomya putoria* | Rio de Janeiro - RJ | Dry |
| Cp7 | *Chrysomya putoria* | Rio de Janeiro - RJ | Dry |
| Cp8 | *Chrysomya putoria* | Rio de Janeiro - RJ | Dry |
| Cma1 | *Cochliomyia macellaria* | Alegre - ES | Alcohol 70 ºG.L. |
| Cma2 | *Cochliomyia macellaria* | Alegre - ES | Alcohol 70 ºG.L. |
| Cma3 | *Cochliomyia macellaria* | Alegre - ES | Alcohol 70 ºG.L. |
| Cma4 | *Cochliomyia macellaria* | Alegre - ES | Alcohol 70 ºG.L. |
| Cma5 | *Cochliomyia macellaria* | Alegre - ES | Alcohol 70 ºG.L. |
| Cma6 | *Cochliomyia macellaria* | Alegre - ES | Alcohol 70 ºG.L. |
| Cma7 | *Cochliomyia macellaria* | Alegre - ES | Alcohol 70 ºG.L. |
| Cma8 | *Cochliomyia macellaria* | Alegre - ES | Alcohol 70 ºG.L. |
