# Supplementary material for: Using high-resolution melting to identify Calliphoridae (blowflies) species from Brazil
Source: PeerJ. 2020 Nov 30;8:e9680. doi: 10.7717/peerj.9680 (PMC7713596; doi:10.7717/peerj.9680)
Supplement: Supplemental Information 2 [file peerj-08-9680-s002.docx]

**Supplementary Table 2.** Examples of COI sequences accession numbers downloaded from BOLD Systems and Genbank used for primer design in this work.

| **Species** | **BOLD ID** | **GenBank**  **Accession number** |
| --- | --- | --- |
| *Chrysomya megacephala* | DIQTB334-11 | * |
| *Chrysomya megacephala* | DIRTT059-11 | KC617813 |
| *Chrysomya megacephala* | FFECU065-14 | * |
| *Chrysomya megacephala* | GBDP13130-13 | KC855272 |
| *Chrysomya megacephala* | GBDP14090-13 | JX430024 |
| *Chrysomya megacephala* | SYC7408-14 | KX052071 |
| ***Chrysomya megacephala*** | **GBMIN18761-13** | **JQ246662** |
| *Chrysomya albiceps* | GBDP16296-15 | KJ394508 |
| *Chrysomya albiceps* | GBMIN18792-13 | JQ246659 |
| *Chrysomya albiceps* | NICC032-13 | KF919012 |
| *Chrysomya albiceps* | NICC037-13 | KF919017 |
| *Chrysomya albiceps* | CDFD003-12 | JX438026 |
| *Chrysomya albiceps* | FFECU018-14 | * |
| *Chrysomya putoria* | GBMIN34242-13 | FJ195384 |
| ***Chrysomya putoria*** | **GBMIN18790-13** | **JQ246663** |
| *Chrysomya putoria* | GBDP0586-06 | AF295554 |
| ***Cochliomyia macellaria*** | **GBMIN18759-13** | **JQ246666** |
| *Cochliomyia macellaria* | BBDIT930-11 | * |
| *Cochliomyia macellaria* | BBDIT935-11 | * |
| *Cochliomyia macellaria* | FFECU242-16 | * |
| ***Lucilia eximia*** | **GBDP2534-06** | **DQ453491** |
| ***Lucilia eximia*** | **GBMIN18753-13** | **JQ246678** |
| *Lucilia eximia* | BNNR030-11 | JN280698 |
| ***Lucilia eximia*** | **BNNR035-11** | **JN280702** |
| *Lucilia eximia* | BNNR036-11 | JN280703 |
| *Lucilia eximia* | BNNR040-11 | JN280706 |
| *Lucilia cuprina* | GBDP0706-06 | AJ417704 |
| ***Lucilia cuprina*** | **GBMIN18783-13** | **JQ246677** |
| *Lucilia cuprina* | DIQT002-08 | * |
| ***Lucilia cuprina*** | **GBDP12075-12** | **JF928476** |
| *Lucilia cuprina* | GBDP4482-08 | EU418577 |

Sequences highlighted in **bold font** are from Brazil.

*Sequences available only in BOLDSystems.
