# Supplementary material for: Using high-resolution melting to identify Calliphoridae (blowflies) species from Brazil
Source: PeerJ. 2020 Nov 30;8:e9680. doi: 10.7717/peerj.9680 (PMC7713596; doi:10.7717/peerj.9680)
Supplement: Supplemental Information 3 [file peerj-08-9680-s003.docx]

**Supplementary Material 3.** Alignment of some COI sequences used in this work to exemplify how were made the design of primers. The region highlighted corresponding to the amplicon, including HRM primers: A) HRM 82 bp and B) HRM 124 bp.


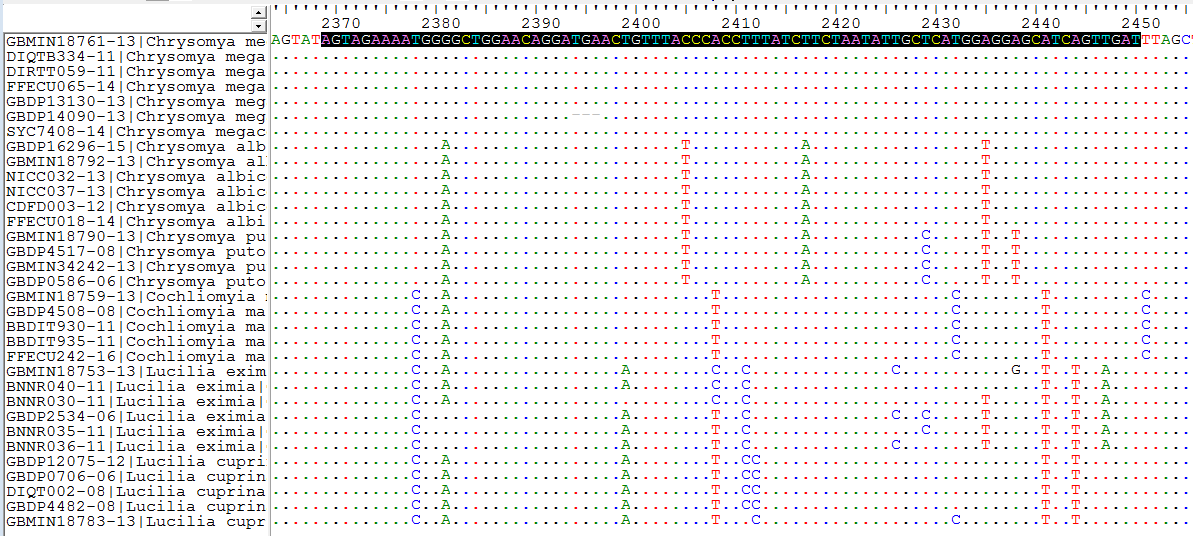


A)


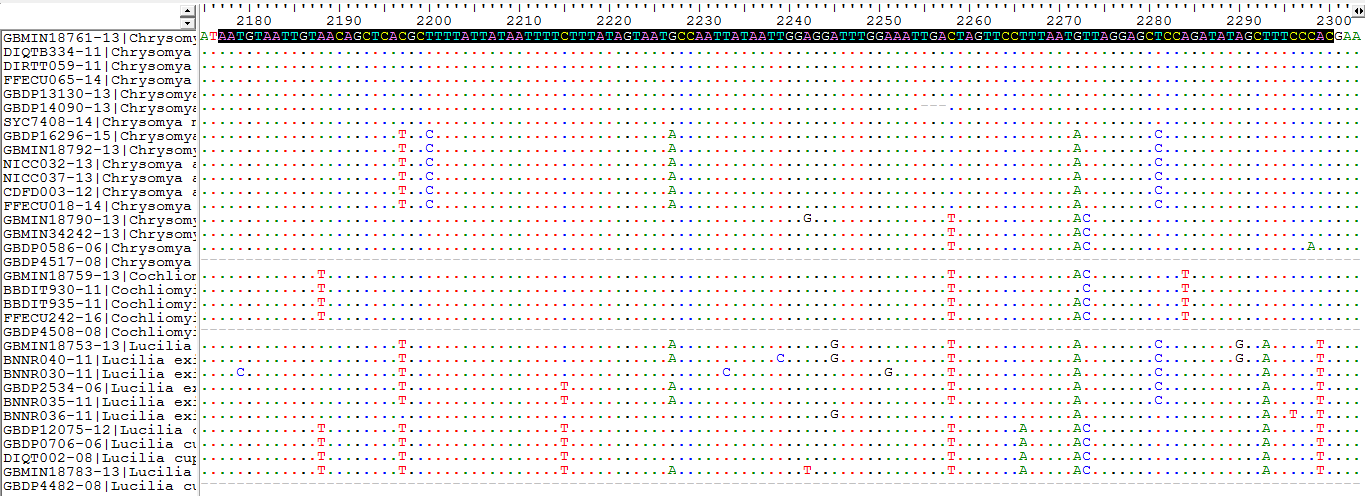


B)

Note: The number of sequences shown in these figures was reduced to fit the size of an image.
